# Chemical Composition of Macroalgae Polysaccharides from Galician and Portugal Coasts: Seasonal Variations and Biological Properties

**DOI:** 10.3390/md21110589

**Published:** 2023-11-10

**Authors:** Sónia P. Miguel, Caíque D’Angelo, Maximiano P. Ribeiro, Rogério Simões, Paula Coutinho

**Affiliations:** 1 CPIRN-UDI/IPG, Center for Potential and Innovation of Natural Resources, Polytechnic of Guarda, Av. Dr. Francisco Sá Carneiro, 50, 6300-559 Guarda, Portugal; spmiguel@ipg.pt (S.P.M.); caique.dangelo@gmail.com (C.D.); mribeiro@ipg.pt (M.P.R.); 2 CICS-UBI, Health Sciences Research Center, University of Beira Interior, Avenida Infante D. Henrique, 6200-506 Covilhã, Portugal; 3 FibEnTech, Fiber Materials and Envornmental Technologies, University of Beira Interior, R. Marques Avila e Bolama, 6201-001 Covilhã, Portugal; rmss@ubi.pt

**Keywords:** algal polysaccharides, seasonal variation, sulphate groups, antioxidant, biocompatibility

## Abstract

Crude polysaccharides extracted from the *Codium* sp. and *Osmundea* sp. macroalgae collected in different seasons (winter, spring and summer) from the Galician and North Portugal coasts were characterised, aiming to support their biomedical application to wound healing. An increase in polysaccharides’ sulphate content was registered from winter to summer, and higher values were obtained for *Osmundea* sp. In turn, the monosaccharide composition constantly changed with a decrease in glucose in *Osmundea* sp. from spring to winter. For *Codium* sp., a higher increase was noticed regarding glucose content in the Galician and Portugal coasts. Galactose was the major monosaccharide in all the samples, remaining stable in all seasons and collection sites. These results corroborate the sulphate content and antioxidant activity, since the *Osmundea* sp.-derived polysaccharides collected in summer exhibited higher scavenging radical ability. The biocompatibility and wound scratch assays revealed that the *Osmundea* sp. polysaccharide extracted from the Portugal coast in summer possessed more potential for promoting fibroblast migration. This study on seasonal variations of polysaccharides, sulphate content, monosaccharide composition and, consequently, biological properties provides practical guidance for determining the optimal season for algae harvest to standardise preparations of polysaccharides for the biomedical field.

## 1. Introduction

Macroalgae are a valuable source of polysaccharides (PSs) with critical industrial applications, namely, in the biomedical field, due to the broad range of biological activities. Skin benefits of macroalgae are mainly related to antiaging, collagen boosting and photoprotective action [1]. The wound healing potential of macroalgae PSs to develop wound dressings has been recently reviewed by Andryukov et al. [2].

These biological properties are markedly related to the physicochemical composition, structure, molecular weight, and sulphation pattern. The seasonal and geographic variation of environmental factors, which determine the macroalgae properties, has been related to changes in photosynthetic rates and, consequently, the carbon available for PS biosynthesis; thus, the variability of macroalgae properties is the main challenge to industrial exploitation for applications considering the requirements for high-quality standards. Also, the biochemical profile of macroalgae PS is strongly influenced by these environmental factors [3]. As for the biomedical properties of macroalgae PS, they can be directly affected by their composition, which can vary according to the season and collection site. So, it is highly important to study and identify the optimal harvesting period to ensure a consistent composition. Several works have reported the biochemical characterisation and bioactivities of different species, namely, *Saccharina longicruris* [4], *Ulva* spp. [5], *Ulva prolifera* [6], *Ascophyllum nodosum* [7], *Laminaria* and *Pelvetia*, and more intensely regarding several species of *Fucus* [7,8,9]. However, the effects of seasons on PSs derived from *Codium* and *Osmundea* species collected from the north of Portugal and Galician coasts have not yet been evaluated. Therefore, this work aims to characterise the polysaccharide composition of these species according to the harvest site and season (winter, spring and summer). Moreover, the biological properties (antioxidant activity, biocompatibility and cell migration ability) were also evaluated. This pioneering work identifies the differences and similarities between the PSs from *Codium* and *Osmundea* harvested from the Portugal and Galician coasts in different seasons regarding the biomedical potential.

## 2. Results and Discussion

### 2.1. Characterisation of Physicochemical Properties of Polysaccharides

#### 2.1.1. FTIR and UV Spectroscopy

Vibrational spectroscopy can reveal detailed information concerning the properties and structure at a molecular level. In the literature, five frequency regions can usually be identified for a vibrational structural analysis of carbohydrates for seaweeds: (1) region of O–H and C–H stretching vibrations at 3600–2800 cm^−1^; (2) region of local symmetry at 1500–1200; (3) region of C–O stretching vibration at 1200–950 cm^−1^; (4) fingerprint or anomeric region at 950–75 cm^−1^; and (5) skeletal region below 700 cm^−1^ [10,11].

**Figure 1:**
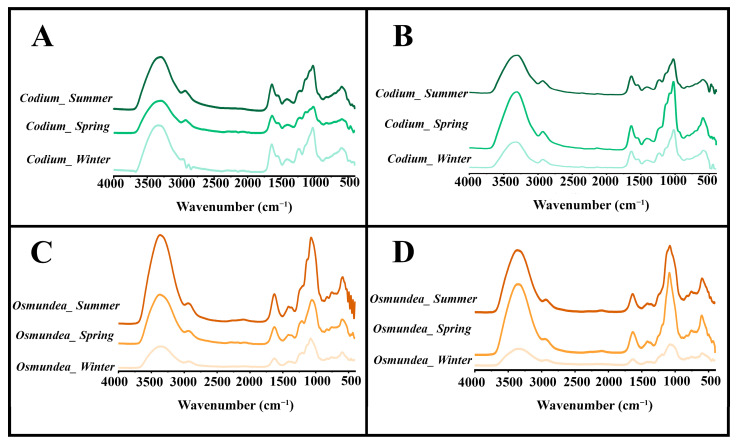
FTIR spectra of polysaccharides extracted from *Codium* sp. and *Osmundea* sp. macroalgae in different seasons (winter, spring and summer) from Galician (**A**,**C**) and Portugal (**B**,**D**) coasts.

From the analysis of the FTIR spectra, the PSs extracted from *Codium* sp. and *Osmundea* sp. from the Portugal and Galician coasts (Figure 1) revealed similar bands to others from macroalgae polysaccharides [11], and phycocolloid standards (carrageenans, agar and alginate) could be observed. A broad band at 3280–3350 cm^−1^ and a weaker signal at 2870–2960 cm^−1^ could be assigned to O–H and C–H stretching vibrations but also to N–H stretching vibrations, respectively.

In turn, the IR absorption bands observed for all the samples between 900 and 1750 cm^−1^ were attributed to the stretching vibration of the C=O group and the presence of sulphate groups for different species. The spectral region between 1200 and 800 cm^−1^ is known as the fingerprinting region of polysaccharides [12]. More yet, the absorbance bands at 1220, 1150 cm^−1^ and 1012 cm^−1^ have been assigned to S=O sulphate esters and to C–O and C–C stretching vibrations of the pyranose and glycosidic ring, respectively [12,13,14]. Moreover, the band near 821 cm^−1^ additionally confirms the sulphate group, as was previously identified for *Codium species* polysaccharides and ascertained to have functional groups like galactose with sulphate and pyruvic acid [14,15]. So, in general, all PS samples presented similar IR absorption bands, with stronger absorption peaks for the PSs extracted from *Osmundea* sp. and collected in summer.

UV–visible spectroscopy has been used to analyse the chromophore groups of atoms characterised by strongly absorbing electronic transitions [16] and revealed that all samples of the PSs extracted from the Portugal and Galician coasts in different seasons (winter, spring and summer) presented a maximum absorption ranging from 260 to 280 nm (Figure S2). Such a feature is commonly attributed to the π−π* electron transitions in aromatic and poly-aromatic compounds found in most conjugated molecules, including proteins. These results indicate that PS can be composed of different compounds (proteins, nucleic acids) that contain multiple UV-absorbing groups [16,17]. Further, it is also possible to verify that such maximum absorption is more pronounced on PSs of *Osmundea* sp. collected in summer on the coast of Portugal. Once more, the locality and season of algal collection has an influence on the physicochemical profile of PSs. Such variation is expected since the protein content changes with the harvesting period due to the variation of environmental parameters like light, temperature and salinity that influence the protein synthesis [18,19].

#### 2.1.2. Surface Charge and Sulphate Groups

**Figure 2:**
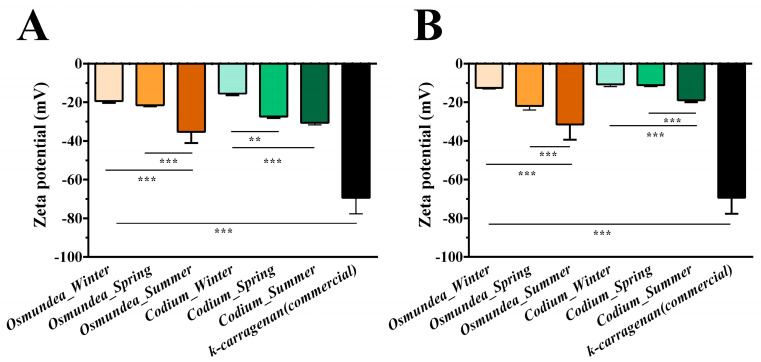
Determination of the surface charge of the polysaccharides extracted from *Codium* sp. and *Osmundea* sp. collected in different seasons (winter, spring and summer) from Portugal (**A**) and Galician (**B**) coasts. The k-carrageenan polysaccharide was used for comparative purposes. Data are presented as mean ± standard deviation (*n* = 5), ** *p* < 0.01, *** *p* < 0.001.

The surface charge of PSs was determined through the DLS analysis to perceive the interactions with biological compounds and structures (Figure 2). Considering the obtained results, all PSs extracted from algal samples possessed an anionic character with zeta potential values between −20 mV and −40 mV. Such an anionic character was also observed on k-carrageenan (which was used as a standard sulphated polysaccharide) and is reported as important to avoid aggregation during blood circulation in the case of PS-based nanoparticles, which can be attributed to the electrostatic repulsion between negatively charged PSs and the cellular surface [20]. On the other side, the negatively charged groups on the surface of PSs could be crucial to produce gels in the presence of divalent cations.

**Figure 3:**
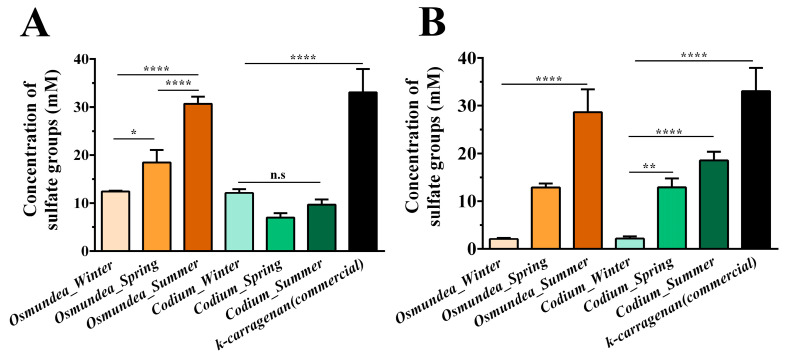
Quantification of the concentration of sulphate groups of the polysaccharides extracted from *Codium* and *Osmundea* species collected in different seasons (winter, spring and summer) from the Portugal (**A**) and Galician (**B**) coasts. The k-carrageenan polysaccharide was used for comparative purposes. Data are presented as mean ± standard deviation (*n* = 5), * *p* < 0.5, ** *p* < 0.01, **** *p* < 0.0001, n.s.—not significant.

Marine algae are the most relevant source of non-animal sulphated PSs, and the chemical structure of these polymers varies according to the algal species and season. PSs extracted from *Osmundea* sp. possessed a higher concentration of sulphate groups than those from *Codium* sp., both for the Portugal (Figure 3A) and Galician coasts (Figure 3B). The samples collected in summer presented a higher amount of sulphate groups in their composition, more evidently on the *Osmundea* sp. -derived PS. The seasonal differences in macroalgae polysaccharides’ biochemical profiles are often attributed to physical factors, such as temperature, salinity, the concentration of nutrients and the amount of sunlight, with a dry mass increase from spring to autumn by about 50% with increased generation of PSs during summer months [4,21] and algae reproduction [22,23,24,25].

It is relevant to notice that the sulphate group composition on PSs is preponderant of their biological properties, namely, antioxidant, anti-inflammatory and anticoagulant [26,27,28]. However, the general correlation between the structure and bioactivity of the sulphated polysaccharides is still not completely established [29].

#### 2.1.3. Molecular Weight

It was noticed that when using a 20 mM phosphate buffer at pH 8 instead of pure water, as eluent, the retention time of the standards slightly increases, indicating a slight decrease in the hydrodynamic volume of the standards with the increase in ionic strength. Similar behaviour occurred for the macroalgae PSs charged and seems to be a natural consequence of the decrease in the hydrodynamic dimension of the polysaccharides under a significant ionic strength, which decreases the electrostatic repulsion [30]. On the contrary, the retention time of the standards decreased when the eluent was changed to NaOH 2.5 mM (pH ≈ 11.4), indicating a slightly higher hydrodynamic volume, which is an expected result considering the higher surface charge and the corresponding electrostatic repulsion increases. The calibration line was adjusted accordingly.

**Figure 4:**
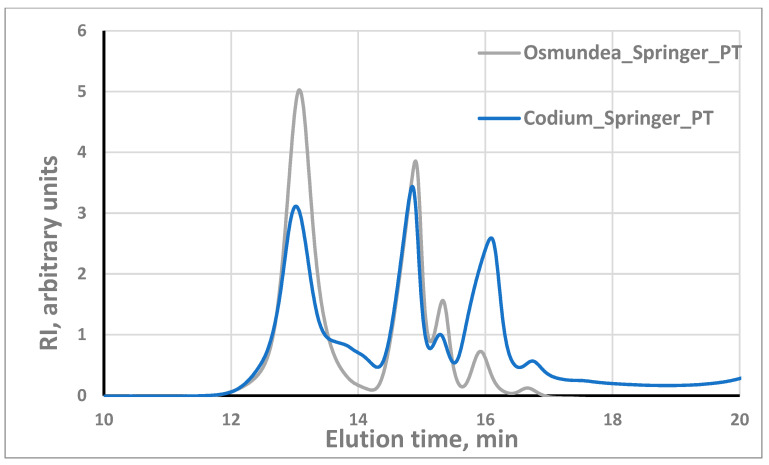
Molecular weight distribution of PSs in *Osmundea* sp. and *Codium* sp. from Portugal coast.

Regarding the algae PSs dissolution, it is worth noting that the dissolution in pure water was relatively low, with dissolution yield in the range of 30–60%, which led us to make the dissolution in a 2.5 mM alkaline aqueous solution, following the work of Wahlström et al. [5]. In fact, under these alkaline conditions, the PS dissolution was very good, with dissolution yields above 85% for both macroalgae PSs. When the alkaline aqueous solution is used as a dissolution solution, the PS concentration should be not higher than 1 g/L to achieve this dissolution. These experimental findings are in agreement with what has been described in the literature for the alginates, which is that solubility can be influenced by factors like pH, concentration, ions in solution and ionic force [31]. Figure 4 illustrates the molecular weight profile of the PSs of the two algae species. The results demonstrate the presence of PS with different molecular weights (several individual peaks at different elution times) in both species. As we can observe in Figure 4, the peaks are practically in the same position (same elution times), suggesting the presence of the same classes of polysaccharides; the differences are at the level of the relative proportion of the different polysaccharides. Regarding repeatability, several GPC runs with the same sample, including with different dilutions, indicated the good performance of the system. A standard deviation of ±0.1 min in the retention time was observed. Unfortunately, it was not possible to associate the several peaks with the corresponding polysaccharides. It should be noted that for assays where higher PSs concentration and water as solvent were used, low dissolution occurred, whereas using water or 20 mM phosphate buffer (pH = 8), some peaks were not separated.

The chromatogram for the PSs from *Osmundea Codium* species when eluted with 2.5 mM NaOH revealed that the most abundant fraction has a retention time of around 13 min (outside the column’s calibration range) corresponding to PSs with a molecular weight probably significantly higher than 150 kDa. However, this conclusion should be taken with caution because the column was calibrated with a neutral PS (dextran), while our PSs have charge, which can change the relation between the retention time and the molecular weight.

Comparing the same species in two environments, the molecular weight distribution (Figure S3) of *Osmundea* sp. harvested from the Galician and north of Portugal coasts, both in spring, suggests that the classes of polysaccharides are the same and only the relative proportion can slightly change. For the *Codium* sp., the classes of PSs are also the same, but the relative proportions are more diverse (Figure S4).

**Figure 5:**
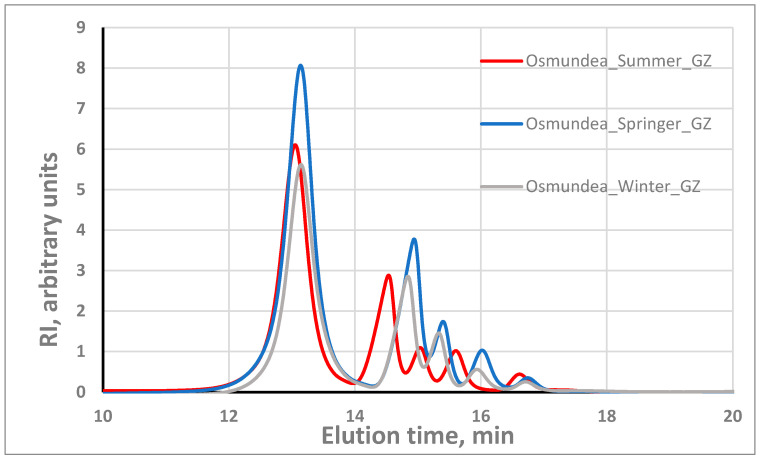
Effect of seasons on the molecular weight distribution of *Osmundea* PSs from Galician coast.

**Figure 6:**
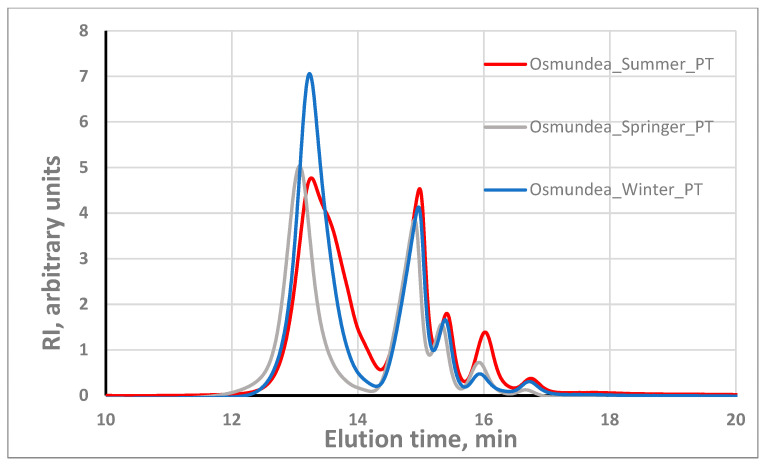
Effect of seasons on the molecular weight distribution of *Osmundea* PS from Portugal coast.

Regarding the effect of season, for the Galician region, there is a trend for a slightly higher molecular weight in summer (Figure 5) for the polysaccharides with molecular weight in the low range. For the Portugal coast, the higher molecular weight seems to occur in spring (Figure 6). For the *Codium* sp. macroalgae, similar behaviour only occurs in summer on the Portugal coast and no significant differences in MWD were observed in Galician. Such variation is expected since the biochemical and rheological profiles change with the harvesting period and are dependent on environmental parameters like light, temperature and salinity, as was described for different species and implies the biological activities [4,32,33].

Although the calibration curve cannot precisely estimate the mean molecular weight of the algae PS because one fraction of the PS exhibits a retention time lower than the standard with the highest molecular weight tested, the calibration curve was established for neutral PS (dextran), the algae PS are charged, and the relative molecular weight can be suggested (Table 1). The results indicate that the values are significantly higher than those reported by Li et al. [34] for PSs from *Codium divaricatum*, after preliminary fractionation, which reported values of 37.9 kDa. The differences obtained in this study can hypothetically be attributed to the extraction methods used and/or the different species under evaluation.

In general, it can be concluded that the algal species presented PSs of different types/classes with substantially different molecular weights, which can be associated with the heterogeneous PSs composition (laminarin, fucoidan, alginate, etc.) of *Codium* sp. and *Osmundea* sp. algae as well as the extraction process used [35].

#### 2.1.4. Monosaccharides Composition

One of the key points in evaluating the monosaccharide composition is the effective hydrolysis of the PSs to monosaccharides before monosaccharides identification and quantification. In the present work, sulfuric acid at 4% (m/m) was used, and a sugar and degradation products recovery between 85% and 95% was obtained, which gives representability to the results. The hydrolysis conditions used led to some degradation products (HMF from hexoses, namely, from galactose, mannose and glucose), but ensured the release of the monomers. Some non-sugar compounds are certainly present in the samples. Based on the monosaccharides used as standards, there are unidentified compounds in the chromatogram. Despite this, it is clear that the algae PSs have significant amounts of xylose, glucose, mannose and arabinose, in addition to galactose, which is the dominant monomer, as presented in Table 2, and similar to other reports for sulphated macroalgae polysaccharides [14].

Li et al. [34] have reported 97.8% of galactose and 2.16% of glucose for the green alga *Codium divaricatum*. Also for *Codium cylindricum* polysaccharides characterisation, results revealed that mannose, galactose and arabinose were the predominant monosaccharides [36], and *Codium fragile* polysaccharides comprised arabinose, galactose and fucose in a 9:2:1 molar percentage [37]. Our results revealed that glucose content (or an unidentified saccharide with the same retention time) and galactose seem to be dominant in *Codium* sp. On the contrary, galactose is dominant in *Osmundea* sp.

Concerning *Osmundea* sp. PSs, the monosaccharides content varied over the year and collection site, in which glucose was higher in spring in Galician coast samples, and galactose, xylose and arabinose in summer, corresponding to the peak of vegetation, as described by Silva and Pereira [38]. This typical composition follows the results obtained for the *O. pinnatifida* extract used in the work of Rodrigues et al. [39], with higher content of galactose and lower quantities of xylose. In general, the results for both algae suggest that galactose content increases significantly in summer.

Similarly, Biancacci et al. [40] reported that the carbohydrate content in *O. pinnatifida* samples varied over the year, with significant differences recorded between the months (highest in May and the lowest in November and January). The results highlight the relevance of such characterisation since both macroalgae PSs showed a diversity of sugars in variable percentages depending on the season and collection site, which is interesting from the perspective of biological activities, and these differences could be attributed to the environmental conditions changes and growth rate [4].

PSs’ composition and molecular weight have a great influence on bioactivity, and important differences are a consequence of different factors like algal genus and species [4,21,33], season [7,41], geographic location [21] and the reproductive stage of the algae [4]. So, it can be assumed that the biological activities are modulated by the biochemical profile of *Codium* and *Osmundea* polysaccharides and dependent on the species, location and collection season; these are further explored.

### 2.2. Determination of the Antioxidant Activity of Polysaccharides

The antioxidant activity of PSs extracted from different algae species collected from the Galician and Portugal coasts in different seasons was evaluated and compared.

Among various biological properties of the macroalgal PSs, the antioxidant properties may have a positive effect on human health since they possess protective properties against damage induced by reactive oxygen species (ROS) [42,43,44]. In particular, when tissue engineering applications are envisioned, the antioxidant property is crucial since it avoids the healing processes’ exuberant and prolonged inflammation response during the healing process. In this way, the antioxidant biological compounds will contribute to the progression of the wound healing process and, consequently, avoid the evolution of chronic non-healing wounds.

**Figure 7:**
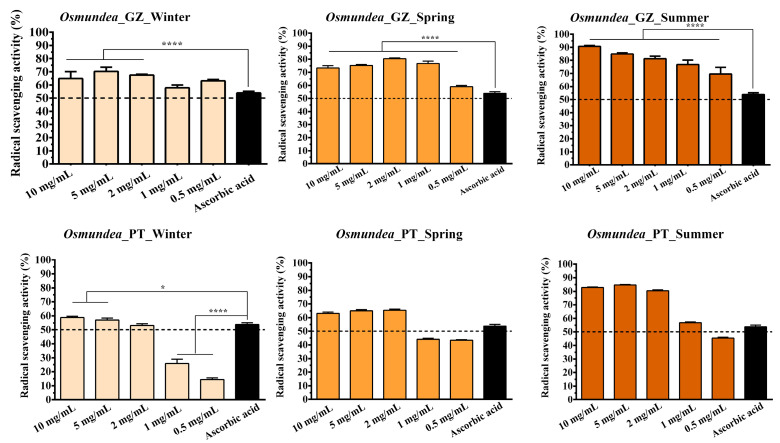
Determination of antioxidant activity through DPPH assay of polysaccharides extracted from *Osmundea* collected from Galician and Portugal coasts in different seasons (winter, spring and summer). Data are presented as mean ± standard deviation (*n* = 5), * *p* < 0.5, **** *p* < 0.0001.

**Figure 8:**
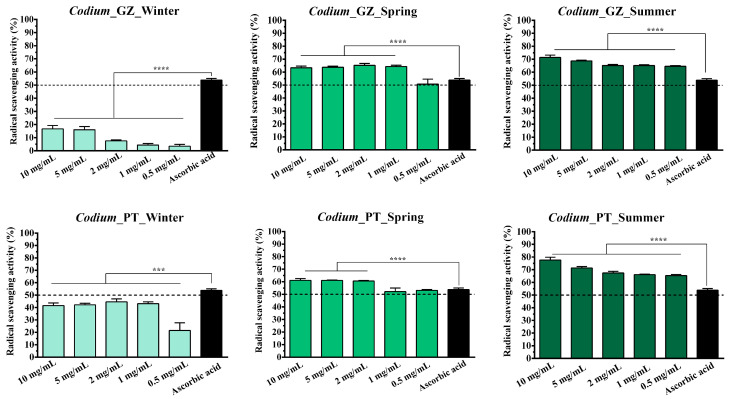
Determination of antioxidant activity through DPPH assay of polysaccharides extracted from *Codium* collected from Galician and Portugal coasts in different seasons (winter, spring and summer). Data are presented as mean ± standard deviation (*n* = 5), *** *p* < 0.001, **** *p* < 0.0001.

So, different concentrations of the *Osmundea* sp. and *Codium* sp.-derived PSs were prepared, and their ability to scavenge the ROS production was evaluated through the DPPH assay. Results confirm that the samples collected in summer presented a higher antioxidant activity for both algal species (Figure 7 and Figure 8). In contrast, lower values were registered for the samples collected in winter. Further, the radical scavenging activity values increased proportionally to the PSs concentration in all samples tested. Such results corroborate the results obtained for the sulphate group’s content since the PSs from summer also possessed a higher concentration of sulphate groups.

Different studies have also demonstrated that the antioxidant activity of PS isolated from seaweeds could be affected by the sulphate content and molecular weight, where a positive correlation between sulphate content and antioxidant activity is observed [42,44,45]. For example, Wang et al. evaluated the scavenging superoxide anion, hydroxyl radical and chelating ability, and the obtained results demonstrated that the reducing power increased with the sulphate contents of extracted algae compounds [42]. Similarly, Ma et al. verified that all the PSs exhibited the DPPH’s concentration-dependent radical scavenging capacity. A higher concentration of PSs indicated a higher radical scavenging rate [46].

It has been appointed that the PS sulphate groups could activate the hydrogen atom of the anomeric carbon, affecting the strong hydrogen-atom-donating ability [47]. However, it is important to notice that other compounds, like low-molecular-weight PSs, pigments, phenolic compounds, and proteins, could also contribute to antioxidant activity [48,49,50].

When comparing the results obtained for *Osmundea* sp. and *Codium* sp., in the samples collected in winter, the difference between samples is noticeable. The differences are not significant between samples collected at both collection sites (Galician and Portugal coasts). Again, the changes in climatic conditions also affect the marine environment, compromising the biochemical parameters of seaweed such as growth and biomass production; nitrogen, phosphorus and potassium contents; photosynthetic pigments; and antioxidant activities [51,52,53].

Furthermore, higher percentages of radical scavenging activity were registered for the *Osmundea* sp.-derived samples, which can also be explained due to the higher content of the mannose and galactose front to *Codium* sp. PSs. Such a hypothesis has been reported in the literature, where the antioxidant activities can also be attributable to the galactose, fucose and mannose contents [54,55].

Considering the obtained results, the PSs extracted from algae collected in summer that present higher sulphate content and antioxidant activity at two different concentrations (5 mg/mL and 2 mg/mL) were selected for the in vitro evaluation of biological properties.

### 2.3. Characterisation of Biological Properties of Polysaccharides in Contact with Fibroblasts

The algae PSs are an excellent option for biomedical applications since these natural compounds possess attractive biological properties, as already previously highlighted by Costa et al. [29] and Wijesekara et al. [43]. So, in this work, the biocompatibility of the algal PSs was evaluated through an MTT assay. For that, the algal PS collected in summer was selected (due to the results obtained in previous assays), and two concentrations (5 mg/mL and 2 mg/mL) were evaluated through an MTT assay.

**Figure 9:**
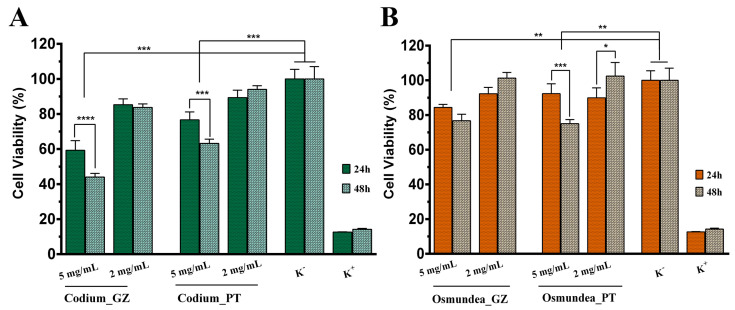
Characterisation of cell viability of fibroblasts in contact with polysaccharides extracted from *Codium* sp. and *Osmundea* sp. collected from Galician and Portugal coasts in summer. Data are presented as mean ± standard deviation (*n* = 5), * *p* < 0.5, ** *p* < 0.01, *** *p* < 0.001, **** *p* < 0.0001.

Concerning the different species tested (*Codium* sp. and *Osmundea* sp.), the PS extracted from *Osmundea* sp. presented higher cell viability percentage values in both concentrations (as shown in Figure 9). At 5 mg/mL of *Codium* sp., the polysaccharide collected from the Galician coast was cytotoxic, with cell viability values lower than 70%. In contrast, the *Codium* sp. from the Portugal coast at this concentration also promoted a decrease in cell viability values after 48 h of incubation.

On the other hand, the *Osmundea* sp.-derived PS presented good cell viability, being more suitable for the concentration of 2 mg/mL of Osmundea_PT.

Our results highlight the promising potential of algal PS for biomedical applications, namely, in tissue engineering. In this field, it is urgent to use natural compounds to produce matrices that promote the healing process, avoiding synthetic, toxic and non-degradable materials. Several reports have reviewed the biological properties of algal PS and their potential for tissue engineering applications [56,57,58,59].

**Figure 10:**
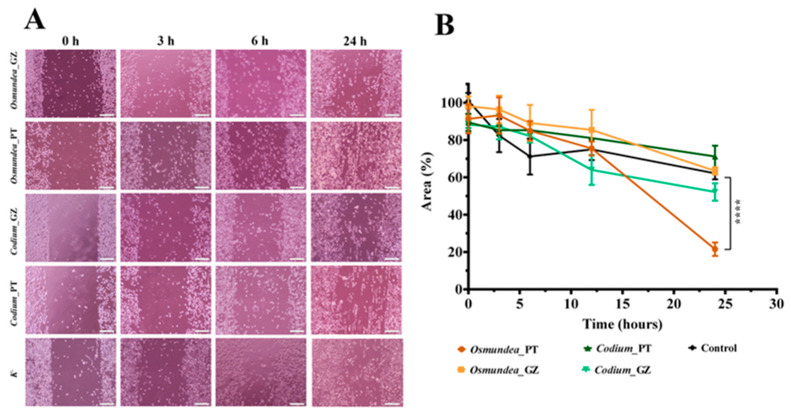
Cell migration response to the different algal polysaccharides. (**A**) Fibroblast migration in the presence of 2 mg/mL of polysaccharides derived from *Osmundea* sp. and *Codium* sp. of Galician and Portugal coasts, and only culture medium (control). (**B**) Effect of the different algal polysaccharides on the migratory activities of fibroblasts in the scratch assay. Data are expressed as a percentage of cell area compared to the control. Scale bar: 200 µm. Data are presented as mean ± standard deviation (*n* = 5), **** *p* < 0.0001.

Considering the results obtained in the MTT assay, the concentration of 2 mg/mL of algal PS was selected to evaluate its ability to induce the cell migration of fibroblast cells. So, a scratch assay was performed, where the periodic microscopic images were taken, and the wound area was calculated during 24 h of incubation (Figure 10). It is perceptible that all samples encouraged the migratory ability of the fibroblast cells, being more evident for the *Osmundea* sp. polysaccharides of the Portugal coast. Such results agree with the previous results, where this sample exhibited a higher percentage of mannose, sulphate groups, antioxidant activity and cell viability percentage. So, it is possible to conclude that PSs’ structural and physicochemical features predict their biological effects, which prompted the cell migration.

The stimulatory effect of algal PS on cell migration was also verified by other authors, appointing that the PS could activate the PI3K/aPKC signalling pathway, promoting cell migration after wounding [60,61].

Altogether, these results demonstrate the potential of algal PS to improve the wound healing process by promoting fibroblast migration, adhesion, and proliferation. Such an event is crucial during skin injury treatment since the fibroblasts are responsible for producing ECM compounds and secreting growth factors focused on the restoration of damaged tissue.

## 3. Materials and Methods

### 3.1. Materials

Dimethyl sulfoxide (DMSO) was purchased from Appli Chem. Panreac (Barcelona, Spain). Ascorbic acid, sodium hydroxide (NaOH) and ethanol were acquired from José Manuel Gomes dos Santos (Odivelas, Portugal). Dulbecco’s modified Eagle’s medium (DMEM)-F12, dextran, ethylenediaminetetraacetic acid (EDTA), foetal bovine serum (FBS), phosphate-buffered saline (PBS) and trypsin were purchased from Sigma-Aldrich (Sintra, Portugal). Normal human dermal fibroblast (NHDF) cells were acquired from PromoCell (Labclinics, S.A., Barcelona, Spain). The 3-(4,5-dimethyl-2-thiazolyl)-2,5-diphenyl-2H-tetrazolium bromide (MTT) was bought from VWR. k-carrageenan was acquired from Quimigen (Alverca do Ribatejo—Portugal).

### 3.2. Methods

#### 3.2.1. Extraction of Polysaccharides from Macroalga *Codium* sp. and *Osmundea* sp.

*Codium* sp. and *Osmundea* sp. macroalgae samples were collected from the Galician and North of Portugal (Viana do Castelo) coasts. Before the PS extraction process, the dry macroalgal samples were subjected to a pre-treatment consisting of the depigmentation and washing of the biomass by immersing the samples into a solution of acetone/ethanol (50:50) and distilled water. Then, the freeze-drying and milling process was performed to obtain dried biomass with the highest surface-to-volume ratio during the latter extraction procedures.

After that, the dried algal samples were re-suspended in distilled water in the ratio of 1:60 (*w*/*v*), and the PSs were extracted through three successive cycles of autoclave (120 °C for 30 min). Then, the extract was centrifuged at 3040G to remove residues, and the supernatant was used to precipitate the PS. The precipitation of PS was achieved with the addition of a triple volume of 96% ethanol, and the precipitate was centrifuged at 3000 rpm, dissolved in distilled water and freeze-dried.

#### 3.2.2. Characterisation of the Physicochemical Properties of Extracted Polysaccharides

##### FTIR, UV Analysis, Surface Charge and Sulphate Groups Determination

The Fourier Transform Infrared (FTIR) spectra were acquired on a Nicolet iS10 spectrometer, with a 4 cm^−1^ spectral resolution from 600 to 4000 nm (Thermo Scientific Inc., Waltham, MA, USA). In turn, the Zetasizer Nano ZS equipment (Malvern Instruments, Worcestershire, UK) was used to determine the charge of PS.

The characteristic UV spectrum of PS was also acquired using the Thermo Scientific Multiskan GO UV/Vis spectrophotometer.

The determination of sulphate groups in PS samples was achieved by barium chloride-gelatin turbidity method, as described in the literature [62,63]. In brief, the precipitation solution was prepared by mixing 0.3% gelatin solution, BaCl_2_ and HCl. After that, about 0.20 mL of polysaccharide solution (1.0 mg/mL) was added to the precipitation solution (40 µL), and the reaction occurred for 10–20 min. A blank was prepared with 0.2 mL of water instead of polysaccharide solution. The released barium sulphate suspension was measured at λ = 420 nm by UV–VIS spectrophotometry using barium sulphate as standard. The standard curve was registered with the regression equation of Y = 0.0201 + 0.0402X, *n* = 5, *R*^2^ = 0.99. The resulting turbidity was measured at 420 nm and compared with standard solutions.

##### Molecular Weight of Polysaccharides

The extracted PS were dissolved in a 2.5 mM NaOH aqueous solution (UHPLC-MS water grade, Carlo Erba, Emmendingen, Germany) at 40 °C in an ultra-sound bath (280 W), turning it on occasionally, and analysed in a high-performance liquid chromatography (HPLC) system composed of an Accela pump 600, Accela Autosampler, Accela PDA detector (Thermo Scientific) and a Refractomax520 (ERC, IDEX Health & Science, Middleboro, MA, USA). After filtration in a 0.45 µm syringe filter, the samples were injected in the Ultrahydrogel TM 250 column (1000–80 kDa) (Waters), using UHPLC-MS water or a 2.5 mM NaOH aqueous solution as eluent, at a flow rate of 400 µL/min. The column was maintained at 50 °C. Dextran (Sigma-Aldrich) with 10 kDa, 19 kDa, 55 kDa, 71 kDa and 150 kDa, and xylose, xylotriose and xylohexase (Megazyme, Wicklow, Ireland), were used as standards, and a calibration line was established between the dextran molecular weight and the elution time in the column (Figure S1). Using Dextran 10 kDa and 150 kDa, a linear correlation was obtained between the dextran concentrations, in the range 0.25 g/L and 2 g/L, and the integral area of the refraction index signal. Using the integrated area of the algae PS samples, an estimate of the dissolved/recovered (detected in the RI) PSs was obtained.

##### Neutral Sugars Composition

The extracted PS were hydrolysed and analysed following the analytical procedures from the National Renewable Energy Laboratory (NREL, Golden, CA, USA) [64], with the necessary adaptions. In brief, 300 mg of sample was submitted to a 4% (m/m) sulfuric acid hydrolysis during 1 h at 121 °C. The neutral sugars, organic acids and carbohydrate byproducts (furfural and hydroxymethylfurfural, (HMF)) in the hydrolysates were analysed using the HPLC system described above. A Rezex ROA (Phenomenex^®^, Torrance, CA, USA) organic acid column was used, with 0.005 N sulfuric acid ultra-pure water as eluent. The column was maintained at 60 °C, and the flow rate was 400 µL/min. Cellobiose, glucose, xylose, mannose, galactose, arabinose, rhamnose, glucuronic acid, formic acid, furfural and hydromethylfurfural (HMF) were used as standards. In this column, xylose, mannose and galactose are eluted at the same retention time, which does not enable their separation but has the advantage of analysing the samples without post-treatment procedures. To quantify the xylose, mannose and galactose separately, the sulfuric acid hydrolysates were neutralised with sodium carbonate to attain neutral pH. After sedimentation, the supernatant was injected into another column, a Rezex RPM-monosaccharide Pb^2+^ (Phenomenex^®^) column, using UHPLC-MS water as eluent. The same neutral sugars were used to calibrate the system. The reported values are the average of two hydrolyses and at least two HPLC analyses for each sample and present a coefficient of variation between 5 and 10%, respectively, for the majority and minority components.

#### 3.2.3. Determination of the Antioxidant Activity of the Polysaccharides

The antioxidant activity of PS was investigated using the 2,2-diphenyl-1-picrylhydrazyl (DPPH) radical scavenging assay, adapting the protocol previously described by Miguel et al. [65]. Briefly, different concentrations of PS were incubated in a DPPH solution (100 μM in methanol). Ascorbic acid at a concentration of 10 µg/mL was used as a positive control. After 30 min of incubation in the dark, the absorbance of each sample was read at 517 nm using a microplate reader (Thermo Scientific Multiskan GO microplate spectrophotometer). DPPH degradation was assessed using the following equation:

$$DPPH\;scavenging\left(\%\right)=\frac{A_{B}-A_{S}}{A_{S}}\times 100$$

A_B_ is the absorbance of the blank sample, and A_S_ is the absorbance of the samples incubated with polysaccharide solutions.

#### 3.2.4. Evaluation of the Biological Activity of the Polysaccharides

The PS’ biocompatibility was evaluated using an MTT assay following ISO 10993-5:2009 (Biological evaluation of medical devices—Part 5: Tests for in vitro cytotoxicity). The Normal Human Dermal Fibroblast (NHDF) cells were cultured in DMEM-F12, supplemented with 10% heat-inactivated FBS and amphotericin B (100 g/mL) in 75 cm^2^ culture T-flasks. Then, the cells were seeded at a density of 5 × 10^3^ cells per well and incubated at 37 °C under a 5% CO_2_ humidified atmosphere.

After 24 h of incubation, the culture medium was replaced by the polysaccharide solutions at different concentrations dissolved in culture medium non-supplemented with FBS (from 10 mg/mL to 0.5 mg/mL). After 24 and 48 h of incubation, the suspension of each well was removed and replaced by a mixture of 50 μL of MTT solution (5 mg/mL), following the incubation for 4 h, at 37 °C, in a 5% CO_2_ atmosphere. After that, the cells were treated with 100 μL of DMSO (0.04 N) for 30 min. The absorbance of each sample (n = 5) was determined at 570 nm using a microplate reader (Thermo Scientific Multiskan GO UV/Vis microplate spectrophotometer). Cells incubated with ethanol (96%) were used as a positive control (K^+^), whereas cells incubated only with a culture medium were used as a negative control (K^−^).

The wound healing scratch in vitro assay was also conducted to determine the PS’ ability to promote cell migration, following the methodology described by Alves et al. [66]. NHDF cells (2.5 × 10^5^ cells/well) were seeded in a 24-well plate with 1 mL of DMEM-F12 until confluence was attained. Then, a linear scratch wound was generated in the monolayer with a sterile 20 µL plastic tip. Any cellular debris was removed by washing the plate with PBS. After, 1 mL of culture medium was replaced by 1 mL of PS dissolved in medium, and then incubated at 37 °C under a 5% CO_2_ humidified atmosphere for 24 h. Cell migration was determined after 0 h, 3 h, 6 h, 9 h, 12 h and 24 h using an Optika inverted light microscope equipped with an Optika B5 digital camera (Bergamo, Italy). The area of cell migration into the wound site was quantified using ImageJ (Scion Corp., Frederick, MD, USA) and presented as a relative migration compared with t = 0, which was considered 100%.

#### 3.2.5. Statistical Analysis

The statistical analysis of the obtained results was performed using a one-way analysis of variance (ANOVA) with the Tukey post hoc test. A *p*-value lower than 0.05 (*p* < 0.05) was considered statistically significant.

## 4. Conclusions

The seasonal variation of PSs from *Codium* sp. and *Osmundea* sp. species of macroalgae, harvested in different seasons from the Galician and North of Portugal coasts, have been studied. The results show distinct differences in the profile and structure of the extracted PSs, both between species and for different periods/seasons and collection sites. Our quantitative analysis suggests that PS structure and biological properties can depend on the seaweed’s genus and biogeographic origin and vary over the collection/harvesting site and season, with the highest content in the summer. These results highlight the need for experimental studies examining the effect of taxon and geographical origin in the biosynthesis of PSs as an alternative for improving both the quality and quantity of these commercial PSs through strain selection.

Concerning the molecular weight, the results suggested that the PSs with higher molecular weight were found in the summer on the Galician coast and in spring on the Portugal coast. In turn, glucose and galactose seem to be dominant monosaccharides in PSs of both macroalgal species.

In addition, it was clearly noticeable that the variations in the physicochemical features are reflected in the biological properties (antioxidant activity, biocompatibility and in vitro induction of cell migration).

This study allows an understanding of the seasonal variation of the *Codium* and *Osmundea* species at different collection sites. The PS extracted from *Osmundea* sp., collected on the Portugal coast in summer, exhibited promising properties for biomedical application purposes. Further, more research is still required to understand the complexity of variation fully. This would include similar studies on the same species harvested from different locations and the effect of the maturity of the species.

## Figures and Tables

**Table 1:** Recovery yield, retention time and weight-average molecular weight of the *Osmundea* sp. and *Codium* sp. polysaccharides with NaOH 2.5 mM as eluent.

Fraction	Yield (%)	Retention Time (min)	MW (kDa)
*Osmundea* sp.—Spring_PT	85.0	13.05; 14.67; 15.17; 15.52; 16.63; 17.28	152
*Osmundea* sp.—Spring_GZ	85.2	13.13; 14.93; 15.40; 16.02; 16.75; 22.10; 22.65	149
*Osmundea* sp.—Summer_PT	83.1	13.27; 14.98; 15.42; 16.02; 16.75; 22.15; 23.05	124
*Osmundea* sp.—Summer_GZ	85.5	13.05; 14.53; 15.03; 15.5; 16.62	186
*Osmundea* sp.—Winter_PT	82.9	13.23; 14.97; 15.38; 15.93; 16.72	139
*Osmundea* sp.—Winter_GZ	95.1	13.15; 14.83; 15.33; 15.93; 16.70	138
*Codium* sp.—Spring_PT	82.3	13.02; 14.85; 15.0; 10.08; 16.73; 21.0; 21.37; 22.0	94
*Codium* sp.—Spring_GZ	62.4	13.08; 14.73; 15.28; 15.95; 16.66; 20.43	169
*Codium* sp.—Summer_PT	95.0	12.98; 14.43; 14.97; 15.5; 16.57	147
*Codium* sp.—Summer_GZ	89.1	13.07; 14.80; 15.30; 16.0; 16.75; 17.72; 22.95	140
*Codium* sp.—Winter_PT	93.3	13.05; 14.65; 15.20; 15.88; 16.68; 20.98	178
*Codium* sp.—Winter_GZ	95.0	13.05; 14.65; 15.25; 15.90; 16.68; 22.95	171

**Table 2:** Monosaccharides composition of *Osmundea* PSs and *Codium* PSs.

	*Osmundea*						*Codium*					
	Spring		Summer		Winter		Spring		Summer		Winter	
	GZ	PT	GZ	PT	GZ	PT	GZ	PT	GZ	PT	GZ	PT
Glucose *	13.6	5.8	4.9	7.9	11.4	7.5	30.6	37.7	35.7	37.3	43.2	41.7
Xylose	9.0	9.0	10.2	6.3	8.4	8.7	4.8	3.4	4.7	2.9	2.5	5.8
Mannose	1.4	1.6	1.0	0.6	0.9	1.0	10.9	26.8	12.9	0.4	4.8	10.7
Galactose	36.9	42.6	39.8	56.0	34.8	38.4	28.0	12.3	25.4	37.6	18.0	17.2
Arabinose	6.1	8.0	9.4	6.1	5.9	5.8	9.7	5.8	6.2	9.1	9.9	8.5
Formic acid	3.70	6.8	4.1	3.3	4.1	6.2	2.7	3.0	4.4	n.d.	12.4	0
Gluc. acid	n.d.	4.9	n.d.	n.d.	6.6	7.3	5.0	3.6	3.5	9.5	n.d.	6.2
HMF	9.4	9.8	9.6	8.7	8.5	7.7	1.9	1.7	2.9	3.1	1.4	1.3
NI	19.9	10.1	21.0	11.1	19.4	17.4	6.4	5.8	4.3	0.0	7.8	7.9

* include cellobiose.

## Data Availability

Data are available upon reasonable request.

## Supplementary Materials

The following supporting information can be downloaded at: https://www.mdpi.com/article/10.3390/md21110589/s1, Figure S1. Size-exclusion chromatography calibration line, using dextran standards and water as eluent; Figure S2. UV–Vis spectrum of polysaccharides from macroalgal species collected in different seasons from Portugal (A) and Galician (B) coasts; Figure S3. Molecular weight distribution of *Osmundea* sp. from Galician and Portugal coasts in spring; Figure S4. Molecular weight distribution of *Codium* sp. from Galician and Portugal coasts in spring.
