# Supplementary material for: Chemical Composition of Macroalgae Polysaccharides from Galician and Portugal Coasts: Seasonal Variations and Biological Properties
Source: Mar Drugs. 2023 Nov 10;21(11):589. doi: 10.3390/md21110589 (PMC10672017; doi:10.3390/md21110589)
Supplement: Supplementary file 1 [file marinedrugs-21-00589-s001.zip › marinedrugs-2694959-supplementary.pdf]

Supplementary Information

1. Results and Discussion

1.1 Calibration curve for size exclusion chromatography

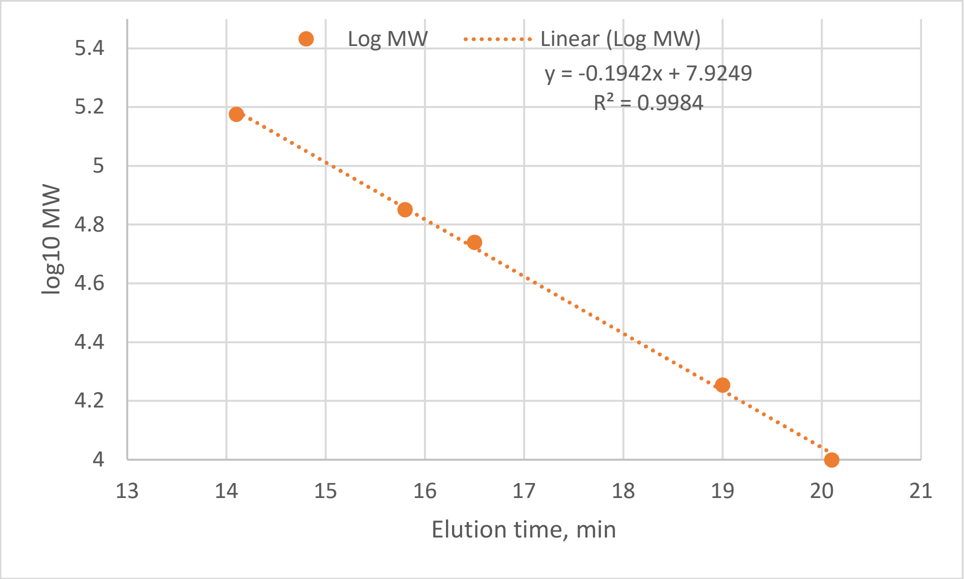

Figure S1. Size exclusion chromatography calibration line, using dextran standards and water as eluent.

1.2 UV-Vis spectroscopy

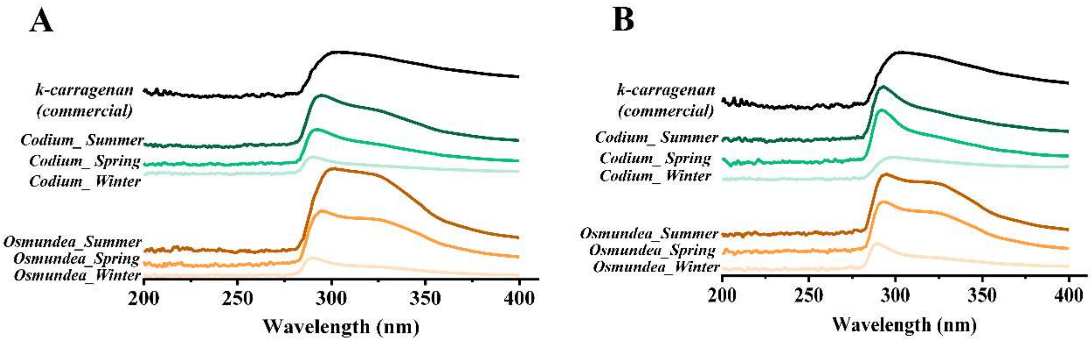

Figure S2. UV-Vis spectrum of polysaccharides from macroalgal species collected at different seasons from Portugal (A) and Galicia (B) coast.

1.3 Molecular weight of polysaccharides

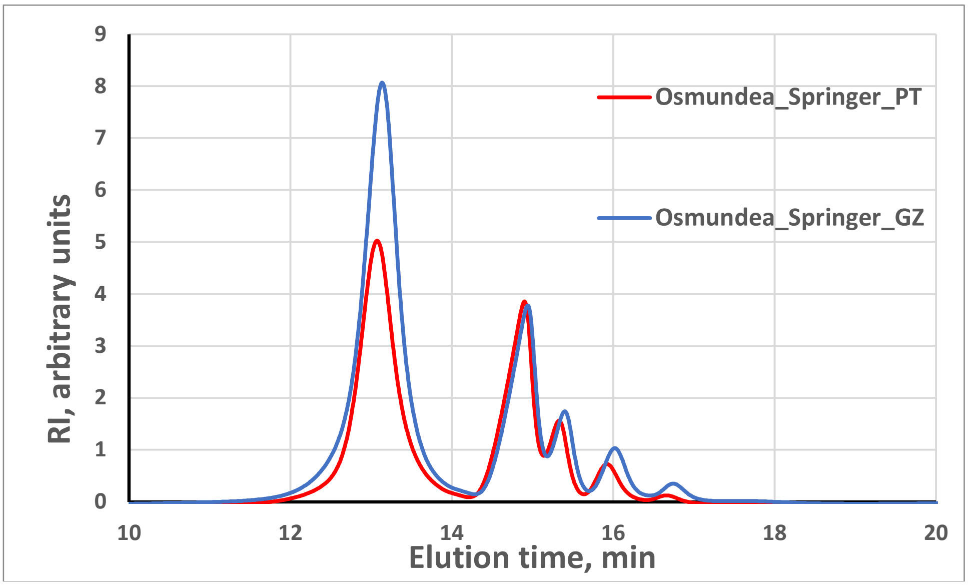

Figure S3. Molecular weight distribution of *Osmundea* sp. from Galicia and Portugal coasts, in spring.

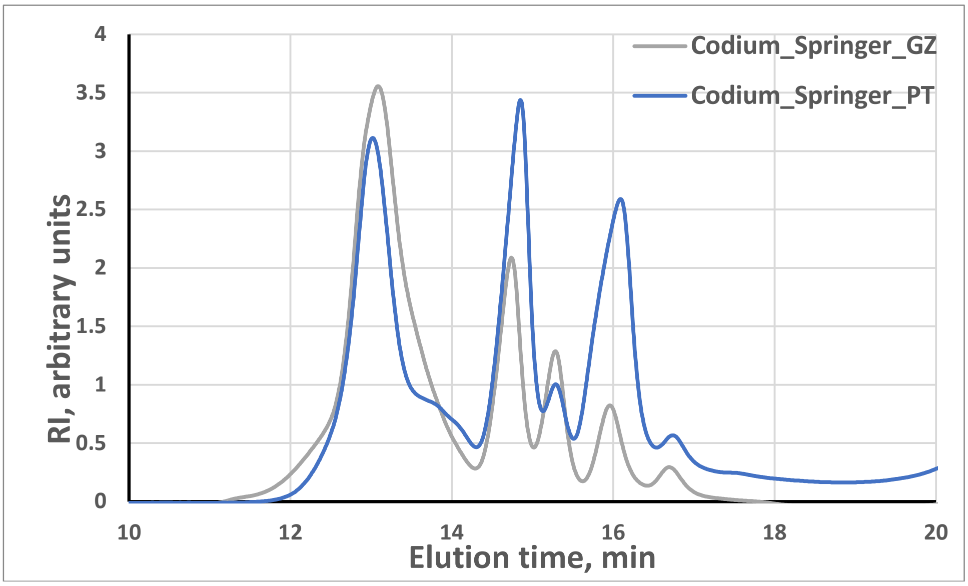

Figure S4. Molecular weight distribution of *Codium* sp. from Galicia and Portugal coasts, in spring.
